# A False-Positive Detection Bias as a Function of State and Trait Schizotypy in Interaction with Intelligence

**DOI:** 10.3389/fpsyt.2014.00135

**Published:** 2014-09-25

**Authors:** Phillip Grant, Mona Balser, Aisha Judith Leila Munk, Jens Linder, Juergen Hennig

**Affiliations:** ^1^Personality Psychology and Individual Differences, Department of Psychology, Justus-Liebig-University Giessen, Giessen, Germany

**Keywords:** schizotypy, psychosis proneness, psychosis continuum, perception, intelligence

## Abstract

Hallucinatory experiences are by far not limited to patients with clinical psychosis. A number of internal and external factors may bring about such experiences in healthy individuals, whereby the personality trait of (positive) schizotypy is a major mediator of individual differences. Psychotic experiences are defined as associating abnormal meaning to real but objectively irrelevant perceptions. Especially, the ambiguity of a stimulus correlates positively with the likelihood of abnormal interpretation, and intelligence is believed to have an important influence and act as protective against clinical psychosis in highly schizotypic individuals. In this study, we presented 131 healthy participants with 216 15-letter strings containing either a word, a non-word, or only random letters and asked them to report, whether or not they believed to have seen a word. The aim was to replicate findings that participants with high values in positive schizotypy on the trait-level make more false-positive errors and assess the role of stimulus-ambiguity and verbal intelligence. Additionally, we wanted to examine whether the same effect could be shown for indices of state schizotypy. Our results support findings that both state and trait positive schizotypy explain significant variance in “seeing things that are not there” and that the properties of individual stimuli have additional strong effects on the false-positive hit rates. Finally, we found that verbal intelligence and positive schizotypy interact with stimulus-ambiguity in the production of false-positive perceptions.

## Introduction

Hallucinatory experiences, especially of an auditory nature, are sometimes erroneously believed to exist only as symptoms of psychosis and, by extension, schizophrenia. A number of studies have shown, however, that such phenomena are not exclusive to clinically psychotic patients, but can be found even in the healthy population; e.g., (1), reports a prevalence of hallucinations of any kind within a large tri-national sample (*n* = 13,057) of 38.7%, whereof no relation to specific pathology was found in more than half of the sample. Visual hallucinations were of considerably higher prevalence than auditory hallucinations (3.2 vs. 0.6%) in the entire sample.

Although a number of clinical and non-clinical factors exist that bring on individual hallucinatory experiences in non-psychotic patients [e.g., stress, caffeine, and the interaction of both; (2)], it has been repeatedly shown that the occurrence of such experiences is mediated by personality traits related to schizotypy [e.g., Ref. (3–5)].

On the phenomenological level, hallucinations have been shown not to actually be seeing or hearing, etc., “things that *are not there*,” but rather – as proposed by Kurt Schneider – interpreting abnormal meaning(fulness) to truly existent but objectively meaningless stimuli [cited from Ref. (6)]. This definition translates well into the current biological model of psychosis and psychosis-proneness by Howes and Kapur (7), who plausibly argue that *abnormal meaning* comes from top-down cognitive explanations of *aberrant salience* attributed (as a function of a dysregulation of dopaminergic neurotransmission) to, essentially, irrelevant stimuli. The authors also clearly argue that individual differences in dopaminergic (dys)regulation in the healthy population also exist. Thus, these differences explain both the appearance of hallucinations in non-psychotic individuals, as well as a possible biological basis of the aforementioned personality trait of schizotypy/psychosis-proneness.

Within the schizotypy-framework as defined by Claridge (8), high levels in schizotypy are not necessarily pathological but potentially beneficial; a concept Claridge refers to as “benign schizotypy.” Especially, when paired with above-average intelligence, which appears to protect high schizotypes against clinical psychosis (9), especially positive schizotypy is associated with creativity (10, 11) and can be found in higher levels in, e.g., artists, novelists, composers, philosophers, etc. [q.v., (9)]. In those cases, where eminent achievers exhibited biographical indices of clinical psychosis, it has to be noted that their major contributions usually preceded their first psychotic episode (12), or that their psychotic conditions had not caused serious disability or, alternatively, interrupted or ended their creative work (13). Thus, it seems that schizotypy (especially positive schizotypy) but not clinical psychosis is linked to benignly being able to see patterns where others see random noise. In the case of signals within noise, highly schizotypic persons have been shown to rely on less information in order to reach conclusions, whereby this *jumping-to-conclusions*-bias does not relate to the quality of conclusions (14–16).

A typical paradigm for the examination of hallucinatory experiences in healthy individuals is the “White Christmas”-paradigm, during which participants are primed with the eponymous Bing Crosby song and then listen to white noise, during which they are asked to press a button every time they believe to hear the song through the white noise. This paradigm has been used repeatedly by others [e.g., Ref. (3, 4, 17)] and has repeatedly shown the number of false-positive hits correlates positively with schizotypic traits. This paradigm is limited, however, as it allows for no modification of the signal-to-noise-ratio (i.e., difficulty), which has been shown to also be important for the occurrence of hallucinatory experiences (18).

Galdos et al. (19) also found a significant increase in the reporting of meaningful speech when, actually, random noise was presented in schizophrenia patients compared to controls. Within the control group, a similar effect was found as a function of positive but not negative schizotypy as well as of familial risk for schizophrenia. Relevant, in this context, are findings by Dubal and Viaud-Delmon (20) wherein self-reported auditory sensitivity was also positively associated with magical ideation.

Regarding other sensory modalities, Fyfe et al. (21) showed that persons high in schizotypic traits were more likely to interpret meaningfulness in the random movement of triangles, and Simmonds-Moore (22) found effects of schizotypic traits to be more pronounced in visual than auditory stimuli. Interestingly, however, in this publication, no main effect of schizotypy was discovered regarding the number of false-positive guesses in both modalities, but high schizotypes showed significantly greater confidence in their false-positive guesses. Similar results have been published by Corlett et al. (23) regarding the confidence in but not the number of false-positive memories. Contrary to these findings, however, Wilson and French (24) found that individuals significantly more often reporting false-positive memories, also had higher scores in schizotypic measures. Also of relevance is the often reported effect that highly schizotypic persons were more likely to display a tendency to say “yes” to ambiguous stimuli in forced-choice paradigms [e.g., Ref. (23, 25, 26)].

The group around Tsakanikos and Reed [e.g., Ref. (18, 27–30)], under the plausible assumption that the link between schizotypy and a false-positive detection bias was not limited to individual sensory modalities (27), thus, created a visual word-detection paradigm. Herein, participants were presented different moving words and non-words and asked to note (a) whether they had seen a “real” word and (b) which word they had seen. Through variations of the paradigm, the instructions or the difficulty of the task, they were able to link positive schizotypy [measured through the *Unusual Experiences*-scale of the O-LIFE (31)] to the tendency to significantly more often see (and also be able to write down) words when in fact non-words were presented. Furthermore, this link was significantly influenced through task-difficulty and instruction-induced expectancy; i.e., schizotypy had the highest influence in instances (a) where a higher rate of true-positive words was expected than was actually presented, (b) where the true-positive rate was actually high, and/or (c) when the task-difficulty was medium. These results suggest that too easy tasks have too high a signal-to-noise-ratio to make “jumping-to-conclusion” necessary and too difficult tasks lack a minimum of actual perceptual input to allow for the emergence of a false-positive detection bias as a function of schizotypy. A significant effect of stimulus-ambiguity was also reported by van Elk (26).

There are, however, some factors that were not examined so far: as briefly mentioned above, the role of intelligence (especially verbal intelligence or vocabulary) can be considered a relevant factor that may potentially moderate or mediate the effect of schizotypy on a false-positive detection bias in a word-detection task.

Furthermore, schizotypic features, although believed to be relatively stable (8), actually show a certain degree of intraindividual variation; although influenced by habitual (trait) schizotypy, the situational (state) proneness for psychotic-like experiences is dependent upon other internal and external factors. In other words, the probability of having a psychotic-like or hallucinatory experience may vary within the same individual; e.g., in response to environmental factors like perceived (social) stress [e.g., Ref. (32) or influences of recreational drugs (33)]. Therefore, the influences of state measures of schizotypy may prove better predictors of a false-positive detection bias than scores in trait questionnaires. Moreover, it can be expected that a combination of both aspects (trait and state), comparable to a diathesis – stress model, would explain the highest amount of variance in false-positive detection.

It was our aim in this paper to incorporate the aforementioned variables into a single experiment and to pose the following research-questions:

1. Can we replicate the findings of Tsakanikos; Reed and colleagues (q.v., above) that unusual experiences (UnEx) as an indicator of positive schizotypy is associated with a false-positive detection bias?
2. Is the false-positive detection bias additionally related to state indices of positive schizotypy and what is the role of perceived stress in this context?
3. What are the influences of signal-to-noise ratio, task-difficulty, and verbal intelligence/vocabulary on the false-positive detection bias?

## Materials and Methods

### Sample

The sample consisted of a total of 131 healthy participants (according to telephone and on-site self-report). Hereof, 27 were male and 104 female (aged between 17 and 73; *M* = 27.43; MD = 24; SD = 10.63). Participants were gathered through university adverts, personal communications, and newspaper adverts looking for individuals with telepathic or other extrasensory perceptive experiences. Participants were fully briefed during a telephone interview as well as immediately before the experiment; on both occasions, all participants stated not currently suffering or ever in the past having suffered from a psychological or relevant medical condition.

Research was approved by the ethics committee of the German Psychological Association (Deutsche Gesellschaft für Psychologie, DGPs).

### Self-report measures

Trait schizotypy was assessed using the German version of the Oxford-Liverpool Inventory of Feelings and Experiences [O-LIFE; (34)]; the *Unusual Experiences* (UnEx) scale was used as a measure of positive schizotypy. The O-LIFE consists of 104 items in a yes/no-format. In order to use UnEx as a factor for a multifactorial ANOVA, participants were trichotomized according to the 33rd-percentiles.

State schizotypic or psychosis-like indices were assessed using a translation of the items used by Barrantes-Vidal et al. (32) for experience sampling methodology. These items are based on the Wisconsin Schizotypy Scales (WSS) and, thus, have a slightly different factor-structure than the O-LIFE [q.v., (35)]. We therefore combined items from the “psychotic-like index” with the “paranoid index” to assess state positive schizotypy. Items suited only for use in experience sampling methodology were omitted (e.g., “Since the last beep, I consumed: Food | Caffeine | Medication | Snuff | Alcohol | Cannabis or other drugs”). Additionally, the item “At the moment I am feeling stressed” was added. All state items were coded on a visual analog scale from 0 (not at all) to 100 (completely) and measured immediately before and after the word-detection paradigm. Verbal intelligence/vocabulary was measured using the Mehrfachwahl–Wortschatz–Intelligenz test [MWT-B; (36)]. The MWT-B consists of several sets of five-letter-combinations, whereof one is an actual word. The participant is tasked with identifying the word; the IQ can be extrapolated from the manual depending on the number of true identifications and the individual words that were (not) identified correctly. The MWT-B was specifically chosen, as it measures (verbal) intelligence in a fashion similar to the trials in our paradigm.

All self-report measures were programed as online-versions using the platform suscisurvey.de.

### Word-detection paradigm

The paradigm was programed using Matlab (version 4.0.7 with Psychtoolbox 3.0.9). The paradigm consisted of 216 trials, each preceded by a fixation cross randomly shown between 1 and 2 s. Stimuli consisted of a 15-letter sequence presented in Arial (font size 24; white letters on black background) at a distance of 50 cm between the participant and the computer monitor. Testing was performed on two computers with identical monitors with equal settings; room illumination was kept at a constant level using window blackouts, and participants were alone in the room during the paradigm.

Stimuli were presented for 750 ms each, followed by the question “Did you see a word?” (with yes/no-format). If the participant answered “yes,” a second question “Please type in the word you have seen” appeared. Thereafter, the participants were instructed to return to their upright-seated position and press the space-bar for the next trial to commence. Initially, four instruction trials were presented, the first containing a true word and the second not containing a true word (presented for 5 s), the third and fourth instruction trials again with a true word and no word, but presented for 2 s. These stimuli were not used during the main paradigm.

Since the words were German, they are not presented within the scope of this English manuscript. Interested researchers are, however, encouraged to contact the corresponding author.

Of the 216 experimental trials, 72 contained a true word (of five-letter length), 72 a non-word designed according to the corresponding true word using the non-word generator *Wuggy* (37) and the final 72 stimuli containing neither a true word nor a non-word. The true words were gathered from a list of 98 German five-letter words that fit certain criteria (i.e., they were singular-case common nouns that contained no umlaut). These 98 words were randomly embedded into 10 other nonsense letters and given to a sample of 242 unrelated students during a lecture at the University of Applied Science, Giessen, by the principal author. The student were asked to rate the word from “easy to detect” to “difficult to detect” (5-point Likert scale) without time limit. From this, average difficulties were calculated and the 12 “easiest” as well as the 12 “most difficult” words were chosen for the paradigm at hand. For each of these 24 words, a corresponding non-word was created. Words and non-words were each presented three times, namely once at the beginning, the middle, and the end of the 15-letter stimulus (e.g., **HEART**ZBKMLPTWFG, ZBKML**HEART**PTWFG, and ZBKMLPTWFG**HEART**; English word and bold print used just here for clarity). Presentation-order was randomized for each participant, but each participant was presented all 216 stimuli.

Responses were coded by hand (and double-checked by an independent persons) into either of four categories; namely, true-positive, true-negative, false-positive, and false-negative. With respect to the aim of these particular study questions, the categories other than false-positive as well as the reactions times shall not be used as dependent variables within this publication.

### Statistical analyses

All statistical analyses were performed using SPSS (version 15). Due to reported effects of age and sex on UnEx [q.v., Ref. (31, 34)] respective variance was removed for between-subject analyses using the generalized linear model by saving standardized residuals and assigning these to the dependent variables. A similar approach was used for the false-positive hit rate in case of significant effects of factors not related to our research question (e.g., age, intelligence).

For within-subject analyses (i.e., influences of word difficulty and -position as well as signal-to-noide ratio), a dependent-samples GLM-analysis was performed with raw false-positive hits as dependent variables in a 2 × 3-design (two steps for difficulty; three steps for position). A second dependent-samples GLM-analysis was also performed for the analysis of effects of the signal-to-noise ratio on the false-positive hits. This was operationalized through the trial-condition (word, non-word, random letters); with increasingly fewer semantically interpretable entities (word > non-word > random letters) the signal-to-noise ratio was expected to decline.

For between-subject analyses (i.e., influences of schizotypy), an independent-sample GLM-analysis was performed with the standardized residuals of the false-positive hits (independently of difficulty or position) as dependent variables and the trichotomized UnEx-group as factor.

For the analyses of the effects of state schizotypic indices and stress, linear regressions were performed with the standardized residuals of the false-positive hits (again, independently of difficulty and position) as dependent variables and the positive schizotypy index and the stress-item as regressors.

In case of replication questions (i.e., the effects of trait or state positive schizotypy on the false-positive detection rate), one-tailed testing was called for Ref. (38); for all other analyses, two-tailed testing was performed.

Data were controlled for outliers, whereby for the effects of positive schizotypy on the false-positive detection rate one case was eliminated; for all other analyses, it was not necessary to remove this case.

## Results

Over the total of 216 trials, our 131 participants reported an average of 5.45 (SD = 4.93) false-positive hits. The range was from 0 (four participants) to 37 (one participant), whereby the latter was considered as the aforementioned outlier, as the next highest number of false-positive hits was 26, followed by 19, and then continuously from 16 through 0. The median was four and there were two modes (1 and 3, with 18 participants each); the second highest number of false-positive hits was four (14 participants). This appears to be substantially higher than in comparable studies by Tsakanikos and colleagues [e.g., Ref. (18)].

The first question was, whether state and trait positive schizotypy had significant influences on the false-positive detection rate. For these analyses, the age- and sex-corrected UnEx-scores were used as dependent variables (in the ANOVA, the sample was trichotomized as described above). Dependent variable was the false-positive detection rate, corrected (as described above) for confounding factors (i.e., intelligence, age, etc.).

We found a significant effect of UnEx (positive schizotypy) on the false-positive detection rate [*F*_2, 127_ = 3.01; *p* = 0.027 (one-tailed)], whereby Bonferroni-corrected *post hoc* test showed that this effect was mainly explained by the highest scores in the high schizotypy group compared to the low schizotypy group [*p* = 0.032 (one-tailed)].

The regression of state positive schizotypy was also significant [β = 0.254; corrected *R*^2^ = 0.057; *F*_1,128_ = 8.85; *p* = 0.002 (one-tailed)]. Perceived stress had no significant predictive power regarding the false-positive hits; the interaction with state positive schizotypy, however, was significant (β = 0.231; corrected *R*^2^ = 0.046; *F*_1,128_ = 4.72; *p* = 0.008).

The effects of word-position and word difficulty were analyzed using a within-subjects model with Greenhouse-Geisser correction on the false-positive hits (uncorrected for between-subject confounders). Both main effects as well as their interaction were significant; i.e., word-position (*F*_1.8, 243.46_ = 9.16; *p* < 0.001; partial η^2^ = 0.066), word difficulty (*F*_1,130_ = 20.01; *p* < 0.001; partial η^2^ = 0.133), and interaction (*F*_1.87,243.49_ = 6.49; *p* = 0.002; partial η^2^ = 0.048). Descriptive statistics showed higher false-positive hits with difficult compared to easy words. Regarding word-position, with easy words the rate of false-positive hits was lower when the word was presented at the beginning of the 15-letter string compared to the middle or end. In the hard word category, false-positives were identical when the word was presented at the beginning or end, but higher when the word was presented in the middle. Over all, the highest and lowest false-positive hits were difficult words presented in the middle and easy words presented at the beginning, respectively.

Both main effects as well as the interaction were no longer significant when IQ was introduced into the model as a covariate; thus, suggesting that verbal intelligence moderates the effects of word-position and -difficulty. It has to be mentioned that an interaction between the within- and between-subject factors in one model could not be analyzed, since the within-subject design did not allow for correction of between-subject confounders, whereby the between-subject design necessitated a correction for between-subject confounders. Using between-subject factors as covariates and, apparently, “correcting” for these factors, is, in fact, not a probate method in this case (39).

When positive schizotypy was entered as a covariate, the main effect of difficulty and the interaction between position and difficulty remained significant, albeit with reduced effects sizes (difficulty: *F*_1, 129_ = 5.65; *p* = 0.019; partial η^2^ = 0.042; interaction: *F*_1.88, 241.87_ = 4.27; *p* = 0.017; partial η^2^ = 0.032). Additionally, although the main effect of position was no longer significant, there was a significant interaction between position and positive schizotypy (*F*_1.82, 234.75_ = 3.327; *p* = 0.042; partial η^2^ = 0.025). This effect was the only one that remained significant (with the same effect size), when both IQ and UnEx were simultaneously entered as covariates.

In case of the effect of the signal-to-noise-ratio (operationalized through “condition”: word, non-word, random letters) the main effect was significant (*F*_1.68, 217.84_ = 102.81; *p* < 0.001; partial η^2^ = 0.442), showing similar false-positive hits in the conditions with the highest and lowest signal-to-noise ratio (word and random letters) but an increased detection bias in the non-word condition. The effect remained significant but of reduced size when UnEx, IQ, and both were entered as covariates into the model (respectively: *F*_1.69, 218.1_ = 22.67; *p* < 0.001; partial η^2^ = 0.149, *F*_1.68, 216.63_ = 4.34; *p* = 0.019; partial η^2^ = 0.033 and *F*_1.7, 217.07_ = 3.86; *p* = 0.029; partial η^2^ = 0.029). There were no significant interactions between condition and either positive schizotypy or IQ.

## Discussion

We found a significant effect of positive schizotypy on the false-positive detection rate, whereby especially highly schizotypic participants had more incidences of “seeing words that were not there” than low schizotypic individuals. This result is in complete agreement with the findings reported by Tsakanikos and Reed (27, 28). Due to the complexity of the other additionally analyzed factors in this study, we chose not to examine the effects of other schizotypy facets or other performance indices (i.e., true-positives and -negatives as well as false-negatives and reaction time) within the scope of this paper for reasons of brevity.

Additionally, we found that not only trait but also state schizotypy, as assessed through the items used in ESM-studies by Barrantes-Vidal et al. (32), significantly predicted the false-positive hits and interacts with self-reported stress. The latter, in and of itself, did not have predictive power regarding the detection bias in our paradigm. Thus, it would seem that the effects of stress moderate the influence of state schizotypy. This is in line with the supposition that stress (or stressful life events) not only act as facilitators of *inter*individual differences in schizotypy or psychotic-like experiences [e.g., Ref. (40)] and *intra*individual differences [e.g., Ref. 32)] but also that highly schizotypic persons are more susceptible to stress (41).

In within-subject designs, we found significant effects of the difficulty of the presented word and the position thereof as well as the interaction of both of these factors. Presenting words that had been rated as difficult to detect leads to higher false-positive errors than easy words. Furthermore, when words were presented in the middle of the 15-letter string, more false-positive errors were made, independently of word difficulty, than when words were presented at either the beginning or the end of the stimulus. In the interaction, easy words presented at the end of the string lead to the lowest and difficult words presented in the middle of the string to the highest rates of false-positive errors.

Regarding the signal-to-noise ratio, which was operationalized through the task condition (i.e., the relation of semantically interpretable unit to “letter jumble”), we found that the condition with middle signal-to-noise ratio (i.e., non-words) elicited higher false-positive hits than the high or low signal-to-noise ratios (i.e., word and random letters).

These results agree with the proposition by, i.a., Tsakanikos (18) that very easy tasks (e.g., high signal-to-noise ratio, easy words, words that began or ended the 15-letter string) usually require less cognitive “filling in the gaps” and, thus, incur less errors. In the highly difficult conditions, persons would make less false-positive errors as they would have more difficulty to identify potentially semantically relevant units and, thus, be more likely to answer that they had not seen a word. In this case, a follow-up question would be, whether these difficult conditions elicited significantly more false-negative errors. The finding most necessary to discuss, in our opinion, is that in case of the difficult words presented in the middle the high false-positive hits mean that participants made double-errors; i.e., an additional false-negative error, as they had, actually, not identified the presented word correctly. Extrapolated to clinical psychosis, this would be comparable to a situation where a patient, e.g., sees an unknown (but truly existent) face but erroneously interprets this as another person’s face or even something else (e.g., the face of a demon). This would explain anecdotal evidence that hallucinations occur more often at night or in the early evening or morning [(42); q.v., patients’ statements on schizophrenia.com]. Also in healthy individuals it is obviously more common to “see something” at night or during dusk and dawn.

The influence of IQ in this case is highly relevant and, to our knowledge, examined for the first time in research of this kind in healthy individuals. The introduction of verbal IQ as a covariate into the model completely eliminated the main effects of difficulty and position as well as their interaction and considerably reduced the effect size of condition. This can be interpreted along the same line of argumentation as before. The higher the intelligence, the more it is likely that difficulty and position will no longer influence the false- as well as the true-positive detection rates (especially considering that we specifically chose an intelligence test with the MWT-B that extrapolates IQ from a number of tasks inherently similar to our paradigm). In other words, the capability of identifying a word independently of its position or difficulty will increase with the aptitude for this kind of task (as measured through the MWT-B). Therefore, the main effects and the interaction will be most prominent in participants with (relatively) low verbal IQ and, thus, were to be expected to no longer be significant when IQ was entered as a covariate. Regarding the reduction of effect size of condition by IQ, the same explanation as above holds.

Going back to the supposition that intelligence may be protective in high schizotypes regarding their transition into clinical psychosis (9), our findings may help explain this. We chose an IQ-test that consisted of a task specifically measuring the aptitude to what we were also measuring in our paradigm. If one extrapolates to crystalline or g-factor intelligence in general, one would also expect that this will influence stimulus-processing on a broader range of levels. Thus, it should be expected that highly intelligent individuals experience less ambiguity in perceived stimuli and thus require less top-down cognitive “filling in” of gaps and are, therefore, less prone to (especially bizzare) psychotic experiences. Future studies will be necessary, in order to ascertain the verisimilitude of this supposition.

The effects of schizotypy are also of particular relevance, especially within the scope of this paper. In order to explore this further, we performed individual one-tailed *ex post facto* t-tests between the high and low schizotypy groups for each variety of word difficulty, word-position, and task condition in order to assess where the effect of schizotypy was strongest.

These analyses showed that differences were most pronounced in the non-word condition (*T*_82_ = 2.55; *p* = 0.006) with a trend in the word condition (*T*_82_ = 1.5; *p* = 0.068) and no difference in the random letters condition. Similarly, schizotypy did not explain significant differences when difficult words were presented, but there was a trend in the cases of presentation of easy words (*T*_72.74_ = 1.53; *p* = 0.065). With regards to position, words presented in the middle lead to the greatest schizotypy-dependent differences in false-positive hits (*T*_82_ = 1.98; *p* = 0.025), especially if these words were easy words (*T*_64.76_ = 2.31; *p* = 0.012). Interestingly, however, although there were not schizotypy-dependent differences in the false-positive hits when difficult words were presented or when words were presented at the beginning of the 15-letter strings, there was a trend in case these factors were combined; i.e., high schizotypes had tendentially higher false-positive hits in cases were difficult words were presented at the beginning of the stimulus (*T*_82_ = 1.46; *p* = 0.074). All effects were in the expected direction; i.e., highly schizotypic persons always had higher false-positive hits rates than low schizotypic persons.

These results, on the one hand, show that in the non-word condition, where most false-positive errors were made, these can be explained significantly by individual differences in trait positive schizotypy; this is, again, in agreement with Tsakanikos (18) that schizotypy-dependent differences are most pronounced in middle signal-to-noise ratios. The same goes for the differences explained by schizotypy regarding the false-positive reactions to word presented in the middle of a 15-letter string. Furthermore, although difficult words lead to a higher false-positive hit rate in general, highly schizotypic persons showed significantly, and borderline-significantly more errors in easy words compared to low schizotypic persons. It can, thus, be asserted that the effects of schizotypy (although significant) are less pronounced than those of the stimuli. This is in line with the repeated findings that (a) any person, independently of schizotypy, can experience psychotic-like experiences when presented with the “right conditions” and (b) that highly schizotypic persons will be more likely to experiences psychotic-like experiences not only in general but especially in situations were low schizotypic persons will not have a psychotic-like experience.

To summarize, we replicated findings that positive schizotypy leads to a “jumping-to-conclusions” bias and that this bias is most heavily pronounced in reaction to relatively highly ambiguous stimuli. Furthermore, we could show that the effects of schizotypy are considerably less strong than those of stimulus-quality and, thus, that schizotypy explains most variance in those conditions that are not of themselves ambiguous enough to lead to errors in judgment in most persons. In other words, ambiguity increases the amount of errors, but highly schizotypic persons require less ambiguity as a facilitator of a false-positive detection bias than low schizotypic individuals.

We extended upon previous findings by showing that (verbal) IQ significantly moderates both a general detection bias as well as the interactions between said bias and trait schizotypy. Furthermore, we showed that not only trait but also state schizotypy significantly predicted false-positive errors. Although both factors correlated significantly in our study [q.v., (32)], an interaction with stress shows that situational psychosis-like experiences in healthy individuals are not solely a factor of habitual schizotypy.

In conclusion, our results further the understanding that hallucinatory experiences in non-clinical individuals are not only a factor of healthy variations in schizotypy but also depend more heavily on the quality of stimulus-perception and cognition as well as (task-specific) cognitive abilities – as shown by the considerable effects explained through verbal IQ.

Over all, we believe that our study presents a significant add-on to other related findings. We replicate previous results and add relevant information regarding, especially, state schizotypy and intelligence. In the future, different variations of paradigms of this sort may be used and, additionally, combined with imaging and psychophysiological methods (e.g., fMRI and EEG). We are also currently in the process of examining the effects of genetic factors in this relation. It could be expected that specific polymorphisms as well as additive effects of different polymorphisms that are relevant to schizotypy may yield further insight. Preliminary results are highly promising, but we chose not to publish these as yet, due to the fact that the number of participants willing to provide a DNA-sample so far is not large enough to reach the statistical power needed for genetic association studies.

## Conflict of Interest Statement

The authors declare that the research was conducted in the absence of any commercial or financial relationships that could be construed as a potential conflict of interest.
